# Effective Radiotherapy for Bilateral Hip Flexion Dysfunction Due to Vertebral Metastasis at the Origin of the Psoas Major: A Case Report

**DOI:** 10.7759/cureus.97647

**Published:** 2025-11-24

**Authors:** Takuya Sasaki, Yojiro Ishikawa, Satoshi Teramura, Takayuki Yamada

**Affiliations:** 1 Medical Education, Tohoku Medical and Pharmaceutical University, Sendai, JPN; 2 Radiology, Tohoku Medical and Pharmaceutical University, Sendai, JPN

**Keywords:** clinical case report, iliopsoas syndrome, irradiation, radiation and clinical oncology, radiation therapy

## Abstract

Hip flexion dysfunction caused by neoplastic involvement of the psoas muscle is rare, and bilateral cases are exceedingly uncommon. Functional decline may result not only from direct tumor invasion but also from surrounding inflammation and edema. However, reports of rapid functional improvement following radiotherapy are limited. We present a case of a man in his 70s who developed bilateral hip flexion dysfunction following initiation of chemotherapy for esophagogastric junction cancer with multiple liver metastases. Several months later, L2-L4 vertebral metastasis resulted in bilateral hip flexion weakness (Manual Muscle Test (MMT), 3/5), leading to difficulty walking. He had no sensory deficits or bladder/bowel dysfunction, with minimal pain and motor dysfunction being the predominant feature. Imaging demonstrated intermediate-attenuation areas around the vertebral bodies and increased attenuation of the fat planes surrounding the psoas muscles, but no clear evidence of direct muscular invasion. The patient underwent palliative radiotherapy to the L2-L4 region with a total dose of 30 Gy in 10 fractions. Within one week of treatment completion, bilateral psoas strength improved to MMT 5/5, with marked recovery of hip flexion function. Functional improvement was sustained despite only minimal radiological tumor regression, with functional recovery preceding radiological response. This suggests that the primary therapeutic mechanism may have been control of inflammation and edema through the anti-inflammatory effects of radiotherapy. This improvement persisted for at least three months, and the patient died several months later. This case represents an exceptionally rare instance of bilateral hip flexion dysfunction secondary to spinal metastasis adjacent to the psoas origin, showing a clinically meaningful response to radiotherapy. It underscores the importance of muscle-related mechanisms in psoas dysfunction and demonstrates the therapeutic efficacy of radiotherapy in such conditions.

## Introduction

The psoas muscle is the only muscle that connects the trunk to the lower extremities, playing a central role not only in hip flexion but also in postural control and gait [1,2]. As the psoas muscle originates from the anterior lumbar spine, dysfunction of this muscle can contribute to reduced quality of life in patients with malignant diseases, particularly those with retroperitoneal tumors or spinal metastases [3]. Traditionally, hip flexion dysfunction caused by neoplastic involvement of the psoas muscle has been described as malignant psoas syndrome (MPS), a condition characterized by severe pain and limited range of motion [3]. More recently, the concept of malignant hip flexion failure syndrome (MHFFS) has been proposed, characterized primarily by motor dysfunction of the psoas muscle [4]. However, both MPS and MHFFS have been reported almost exclusively as unilateral conditions, and to our knowledge, very few cases of bilateral hip flexion dysfunction have been reported. Consequently, therapeutic approaches have focused primarily on palliative pain management [5], with published cases remaining limited. Here, we report an extremely rare case of bilateral hip flexion dysfunction secondary to lumbar spinal metastasis from esophagogastric junction cancer, which demonstrated rapid and marked functional recovery following radiotherapy. This unique presentation of bilateral dysfunction and prompt functional improvement provides important insights into the pathophysiology of psoas dysfunction and demonstrates the therapeutic potential of radiotherapy in such conditions.

## Case presentation

**Figure 1:**
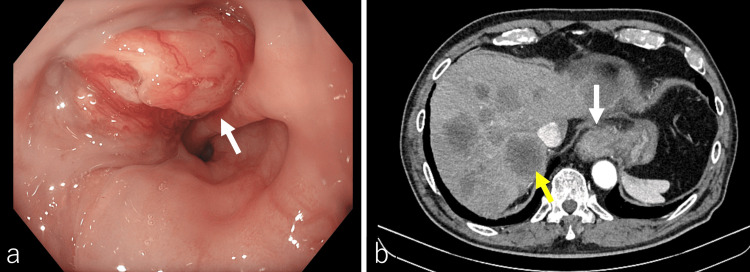
Initial endoscopy and abdominal contrast-enhanced CT Upper gastrointestinal endoscopy revealed a 3-cm tumor at the esophagogastric junction (a, white arrow). Contrast-enhanced CT demonstrated a mass at the esophagogastric junction (b, white arrow) and multiple liver metastases (b, yellow arrow).

The patient was a Japanese man in his 70s with a medical history of hypertension managed with amlodipine 5 mg daily and azilsartan 20 mg daily, arrhythmia, asymptomatic cholelithiasis, and diabetes mellitus that was not receiving specific pharmacological treatment. He had undergone an appendectomy at age 30, had no prior history of malignancy, and was independent in all activities of daily living, maintaining an active lifestyle before this illness. He had no history of tuberculosis. His family history was notable for type 2 diabetes mellitus in his mother. At initial presentation, the patient reported progressive dysphagia. Upper gastrointestinal endoscopy revealed a 3-cm ulcerative tumor at the esophagogastric junction, and biopsy confirmed adenocarcinoma. The histological diagnosis was adenocarcinoma with moderately to poorly differentiated tubular components. Contrast-enhanced CT demonstrated multiple liver metastases, leading to a diagnosis of esophagogastric junction cancer (cT3N1M1, stage IVB) (Figure 1).

Molecular testing revealed human epidermal growth factor receptor 2 (HER2) positivity and Claudin-18 negativity. Mismatch repair proteins (MLH1, PMS2, MSH2, and MSH6) were all preserved, indicating a microsatellite-stable tumor. PD-L1 testing was not performed. ​​​​​Chemotherapy with the SOX regimen (S-1 80 mg/m² daily on days 1-14 and oxaliplatin 170 mg/m² on day 1, repeated every three weeks) was subsequently initiated. Soon after diagnosis, palliative radiotherapy to the primary lesion (30 Gy in 10 fractions, 3 Gy per fraction, one fraction daily, five fractions per week) was administered. Subsequently, three cycles of the T-SOX regimen (SOX plus trastuzumab 8 mg/kg at the first dose, then 6 mg/kg on day 1 every three weeks) were introduced. However, trastuzumab was discontinued due to an infusion reaction, and treatment was continued with S-1 monotherapy (80 mg/m² daily on days 1-28, repeated every six weeks). At the time of initial diagnosis, the patient’s serum tumor marker levels were markedly elevated, with a carcinoembryonic antigen (CEA) of 821 ng/mL and a carbohydrate antigen 19-9 (CA19-9) of 18,573 U/mL. Approximately three months later, both markers had further increased (CEA from 1,656 ng/mL to 2,983 ng/mL; CA19-9 from 28,259 U/mL to 73,863 U/mL), consistent with disease progression on imaging.

On physical examination prior to radiotherapy, bilateral hip flexion weakness (Manual Muscle Test (MMT) 3/5) was observed, resulting in wheelchair dependence due to gait dysfunction (Video 1). Muscle strength was preserved in other regions. At this time, his Eastern Cooperative Oncology Group performance status was 2. There were no sensory deficits or bladder/bowel dysfunction, and pain was minimal, localized to the bilateral hip and lower back regions, with motor dysfunction being the predominant symptom. Pain assessment using the Visual Analog Scale was not recorded. In our case, the lesion at the L2-L4 levels showed osteosclerotic changes with a partial compression fracture, without mechanical pain or significant deformity, corresponding to an estimated Spinal Instability Neoplastic Score of approximately 4-5 points, indicating a stable to indeterminate condition. Therefore, surgical fixation was not considered necessary at that time.

**Video 1:** Clinical findings before radiotherapy Bilateral hip flexion weakness was evident.

**Figure 2:**
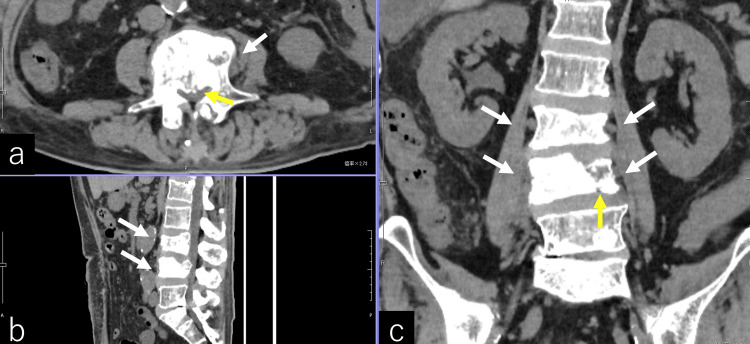
Abdominal plain CT Findings included osteosclerosis of the L4 vertebra (a, white arrow) and spinal canal compression (a, yellow arrow). Sagittal view demonstrated osteosclerosis of L3–L4 (b, white arrows). Coronal view revealed osteosclerosis of the L3–L4 vertebrae with bilateral extension toward the psoas muscles (c, white arrows). A partial compression fracture of the L4 vertebra was also noted (c, yellow arrow).

**Figure 3:**
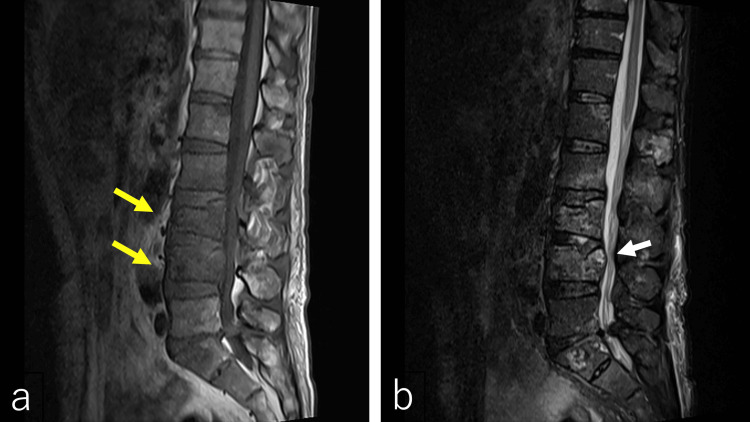
Lumbar spine MRI T1-weighted images showed low-intensity lesions within L2–L4 vertebrae (a, yellow arrows). T2-weighted images demonstrated spinal canal narrowing at the L4 level with anterior displacement of the spinal cord and cauda equina (b, white arrow).

Imaging findings included intermediate-attenuation areas around the vertebral bodies and increased fat plane attenuation surrounding the psoas muscles on CT (Figure 2). MRI revealed low signal intensity within L2-L4 vertebral bodies on T1-weighted images and metastatic tumor components at L4 with posterior extension on T2-weighted images (Figure 3).

**Figure 4:**
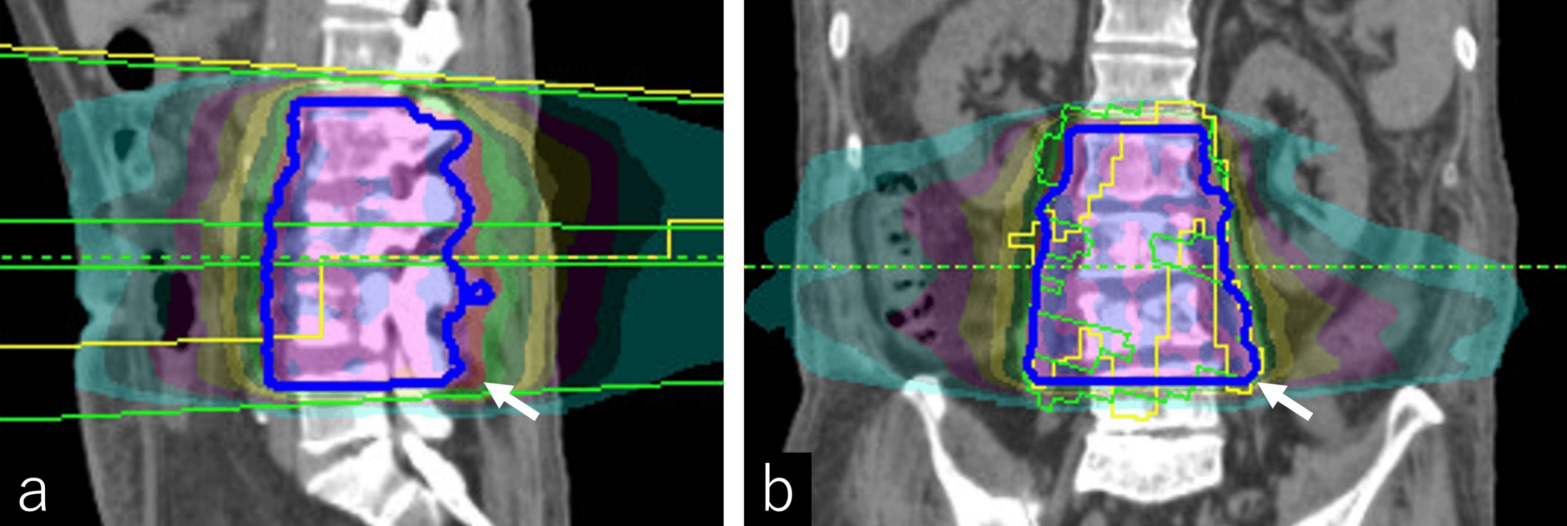
Radiotherapy treatment planning Sagittal (a) and coronal (b) dose distribution images showing the irradiation field to the L2–L4 vertebrae, with white arrows indicating the main distribution area. Treatment planning was performed using RayStation (RaySearch Laboratories AB, Stockholm, Sweden) with a 10-MV photon beam and volumetric modulated arc therapy (VMAT). A total dose of 30 Gy in 10 fractions was delivered to the L2–L4 region. Based on the treatment plan, the mean dose delivered to the bilateral psoas muscles was approximately 25 Gy due to their anatomical proximity to the vertebral metastases.

The metastatic bone lesion originating from esophagogastric junction cancer extended bilaterally in close proximity to the psoas muscle origin; however, a biopsy was not performed. Palliative radiotherapy was indicated for newly developed vertebral metastases at the L2-L4 levels, which caused bilateral hip flexion weakness and difficulty walking. The purpose of radiotherapy was both to relieve neurological compression and to improve lower limb motor function. Palliative radiotherapy was therefore delivered to the L2-L4 region with a total dose of 30 Gy in 10 fractions (3 Gy per fraction, one fraction daily, five fractions per week) (Figure 4).

One week after completion of radiotherapy, the patient’s pain had completely resolved. Bilateral psoas muscle strength improved to MMT 5/5, with marked recovery of hip flexion function. Although wheelchair use was continued due to the patient’s general condition, bilateral motor dysfunction had clearly resolved (Video 2). Before radiotherapy, active hip flexion was limited to approximately 30-45° bilaterally, while passive flexion was nearly full. After treatment, active hip flexion improved to about 90° on both sides, and full passive motion was maintained; however, a detailed orthopedic assessment was not feasible due to advanced disease. The patient was able to stand after treatment; however, because of the risk of falls, wheelchair use was maintained as the primary means of mobility. Independent ambulation could not be achieved owing to disease progression.

**Video 2:** Clinical findings after radiotherapy Hip flexion markedly improved, with restoration of iliopsoas muscle strength to normal levels.

## Discussion

In the present case, there was no clear evidence of direct invasion into the psoas muscle, and imaging findings did not suggest tumor infiltration or abscess formation. Therefore, the pathophysiology was considered to represent reversible functional dysfunction due to inflammation and edema surrounding the psoas muscle origin, secondary to L2-L4 vertebral bone metastases. While Watanabe et al. [6] previously reported functional dysfunction associated with a psoas abscess in a pediatric patient, only a few cases of psoas inflammation with subsequent functional alteration have been reported in the literature. To our knowledge, reports of functional dysfunction caused by inflammatory changes near the psoas origin remain extremely rare. Psoas dysfunction encompasses heterogeneous pathophysiological mechanisms and may result not only from direct tumor invasion but also from surrounding edema and inflammatory responses. MPS has been reported in several cases since its first description in 1990, with the vast majority presenting unilaterally [3,7].

Although one previous case of bilateral psoas involvement has been reported in a patient with advanced bladder cancer who had a poor prognosis and died within eight months [8], no prior reports have described bilateral hip flexion dysfunction secondary to vertebral metastasis from esophagogastric junction cancer. While bone metastases occur in approximately 47% of metastatic bladder cancer cases [9], the incidence of bone metastasis in esophageal cancer, the primary malignancy in our patient, is relatively low at around 7.7% [10]. However, the rarity of the present case is not only related to the low incidence of bone metastasis in esophageal cancer but also to its distinct clinical manifestation, characterized by bilateral hip flexion weakness resulting from metastatic involvement near the origins of both psoas muscles. This combination of anatomical localization and functional impairment underscores the exceptional nature of this presentation.

Another distinctive feature of our case was the clinical presentation. While conventional MPS typically manifests as refractory pain due to direct tumor invasion, our case was characterized predominantly by muscle weakness with minimal pain. Recently, the concept of MHFFS has been proposed, characterized by muscle weakness as the predominant feature [4]. Our patient's clinical course was consistent with MHFFS. In contrast to the unilateral cases reported by Ishikawa [4] and Teramura [11], the present case demonstrated bilateral involvement, representing a novel contribution to the literature. Although imaging demonstrated compression of the cauda equina, several factors argued against a neurogenic etiology: the anatomical mismatch between the nerve roots innervating the psoas muscle (L1-L3) and the level of spinal compression (L3-L4), the absence of sensory deficits or bladder/bowel dysfunction, and the rapid functional recovery following treatment. These findings strongly suggested that the dysfunction was myogenic rather than neurogenic in origin, possibly reflecting myositis of the psoas muscle. Psychological factors such as distress could not be identified in this case.

The most clinically significant finding was the marked functional recovery following radiotherapy that preceded radiological improvement. Kashiwagi et al. [12] reported the efficacy of radiotherapy for intractable pain associated with MPS. Traditionally, the primary objective of radiotherapy for psoas involvement has been pain palliation, with limited emphasis on functional recovery [13-15]. In our case, the functional improvement was most likely attributable to the anti-inflammatory effects of radiotherapy, which reduced intramuscular inflammation and edema [16,17]. While definitive conclusions cannot be drawn from a single case report, this case suggests that early initiation of radiotherapy in patients with neoplastic lesions adjacent to the psoas muscle may contribute to improved functional outcomes. The limitations of this study include its single-case design and the absence of electrophysiological testing to definitively exclude neurogenic components. Future accumulation of similar cases and long-term follow-up studies will be necessary to further elucidate the clinical significance of these findings.

## Conclusions

We report an extremely rare case of bilateral hip flexion dysfunction secondary to spinal metastasis adjacent to the psoas muscle origin, which demonstrated remarkable functional improvement within a short period following radiotherapy. Based on the anatomical pattern of nerve innervation and clinical findings, cauda equina syndrome was excluded, and the impairment was attributed primarily to myogenic dysfunction resulting from either direct tumor extension or secondary inflammation and edema. To our knowledge, this represents the first reported case of bilateral psoas dysfunction in advanced esophageal cancer.

This case demonstrates the potential benefits of early recognition and prompt radiotherapeutic intervention in achieving functional recovery. In addition, this case highlights the importance of a multidisciplinary approach, including radiation oncology, medical oncology, rehabilitation, and palliative care, to optimize both symptom control and functional outcomes in complex metastatic conditions. Such clinical presentations remain underrecognized in clinical practice, and this report may provide valuable diagnostic and therapeutic guidance for clinicians encountering similar cases.
